# Confirmed bacteriological diagnosis and cure of nonsevere gram-positive clinical mastitis cases enrolled in a randomized clinical trial based on results of on-farm culture

**DOI:** 10.3168/jdsc.2024-0560

**Published:** 2024-05-10

**Authors:** Q.K. Kolar, S.M. Godden, R.J. Erskine, P.L. Ruegg

**Affiliations:** 1 Department of Animal Science, Michigan State University, East Lansing, MI 48824; 2 Department of Veterinary Population Medicine, University of Minnesota School of Veterinary Medicine, St. Paul, MN 55108; 3 Department of Large Animal Clinical Sciences, Michigan State University, East Lansing, MI 48824

## Abstract

Our objectives were to describe the confirmed bacteriological diagnosis of mastitis pathogens cultured from cases of nonsevere clinical mastitis (CM) identified as gram-positive (GP) using on-farm culture and to compare differences in bacteriological cure (BC) based on antimicrobial (AM) therapy. Cows that developed nonsevere CM in a single quarter were enrolled in a randomized clinical trial based on microbial growth on GP selective agars. Cows were randomly assigned to receive a once-daily intramammary treatment: 3 d hetacillin (n = 69), 3 d ceftiofur (n = 69), 8 d ceftiofur (n = 70), or to a nontreated group (n = 32). Etiologies were confirmed with MALDI-TOF using frozen duplicate milk samples. The crude proportion of BC was determined using milk samples collected at 14, 21, and 28 d after enrollment for cases caused by streptococci, NAS, *Streptococcus*-like organisms (consisting of *Enterococcus* and *Lactococcus*; SLO), and *Staphylococcus aureus.* Multivariable models were used to determine the effect of AM treatment on BC cure at d 21 for a subset of cases that had complete data for all covariates (n = 88). Growth on GP agar resulted in enrollment of 240 cases with confirmed etiologies distributed as *Streptococcus* spp. (21.7%; n = 52), *Lactococcus* spp. (19.2%; n = 46), NAS (16.3%; n = 39), *Staph. aureus* (6.3%; n = 16), *Enterococcus* spp. (5.0%; n = 12), and others (10%; n = 24). Thirty-five (14.5%) duplicate milk samples collected from enrolled cows had no significant growth in the laboratory and 16 (6.7%) were contaminated. Among sampling dates, combined crude BC for cases caused by target pathogens ranged from 68% to 71%. Crude BC ranged from 58% to 73% for the small group of nontreated cases (n = 12) and 69% to 71% for the combined group of cases that received intramammary AM (n = 113). Among all groups and all follow-up dates, crude BC was least for cases caused by *Staph. aureus* (ranging from 33% to 43%), followed by cases caused by SLO (58%–67%), NAS (79%–80%), and *Streptococcus* spp. (81%–91%). The proportion of BC at 21 d (LSM ± SE) did not vary between all cases that received AM (0.77 ± 0.06) and cases in the nontreated group (0.73 ± 0.16). The odds of BC at 21 d was about 5× greater for cases that occurred in primiparous as compared with multiparous cows and decreased 1.3-fold for each 1 log unit increase in SCC before the case. While this study does not have sufficient power to detect small differences in BC among pathogen groups or treatments, our results demonstrate that CM is caused by a wide diversity of GP bacteria with varying ranges of BC. These results provide justification for inclusion of negative control groups in future studies and confirm that important characteristics such as parity and history of subclinical mastitis influence the probability of BC.

## Highlights

• Lactococci and enterococci accounted for 24% of isolates causing nonsevere clinical mastitis.
• Fifteen percent of duplicate milk samples collected from quarters with gram-positive growth were culture negative.
• Bacteriological cure was similar to previous studies.
• Increased parity and a history of subclinical mastitis reduced odds of bacteriological cure.

## Graphical Abstract

**Figure:**
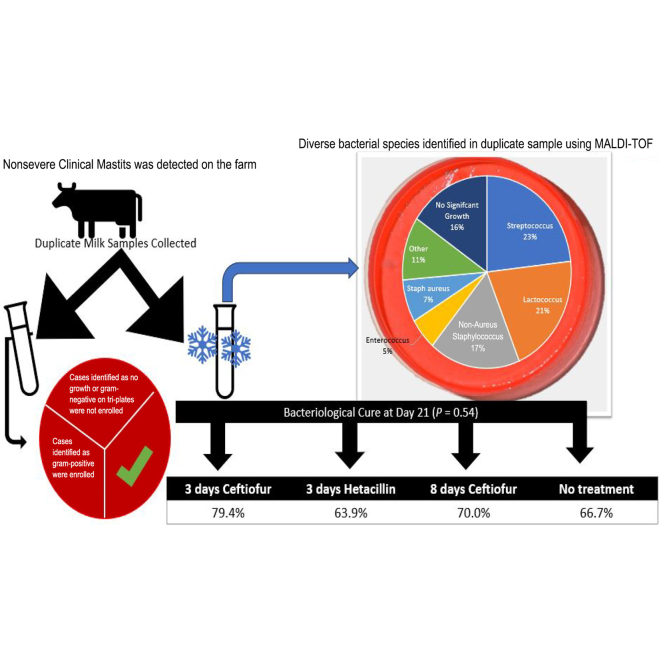
**Summary:** Nonsevere clinical mastitis identified as gram-positive using on-farm culture was caused by a wide range of pathogens. The primary pathogens identified on these farms were *Lactococcus* species. When comparing the bacteriological cure between the 3 different antimicrobial treatments and no treatment, bacteriological cure did not vary between the treated and controls 21 days after case detection. Cows with a greater parity and a history of subclinical mastitis before the enrolled case had reduced odds of bacteriological cure at day 21.

Clinical mastitis (**CM**) is the most frequent disease of dairy cows and the most common reason that antimicrobial (**AM**) treatments are administered to dairy cows (de Campos et al., 2021). In the United States, most cases of nonsevere CM are treated using intramammary (**IMM**) AM (USDA, 2016), and an emphasis on reducing AM usage has led to development of on-farm culture programs (**OFC**) used to guide selective treatment protocols (Lago and Godden, 2018). Selective treatment protocols emphasize use of AM to treat CM caused by streptococci and staphylococci but not for treatment of CM caused by *Escherichia coli*, or chronic infections of *Staphylococcus aureus* (de Jong et al., 2023). Using these criteria, IMM AM therapy is recommended for cases that result in growth of gram-positive (**GP**) bacteria, while AM are not generally recommended for cases that result in growth of gram-negative (**GN**) bacteria nor those that are culture negative when detected (Lago and Godden, 2018).

Whereas OFC programs usually use growth on selective agars to broadly categorize etiologies, diagnostic laboratories use more definitive methods. Use of MALDI-TOF to identify mastitis pathogens has demonstrated that *Lactococcus* spp. comprise a considerable proportion of organisms previously classified as “environmental *Strep*.” (Scillieri Smith et al., 2020). When grown on selective agar, *Lactococcus* and *Enterococcus* (*Streptococcus*-like organisms; **SLO**) are phenotypically indistinguishable from *Streptococcus* spp., and when using OFC, AM therapy would be recommended even though efficacy of IMM AM is unknown for these organisms.

Our objectives were to describe the confirmed bacteriological diagnosis of mastitis pathogens that were identified as GP and received AM therapy based on results of OFC and to compare differences in bacteriological cure (**BC**) based on use of IMM AM. We hypothesized *Streptococcus* and *Staphylococcus* would be the most common etiologies and cases that received IMM AM would have improved BC as compared with cases that did not receive AM.

A negatively controlled, randomized clinical trial was conducted on dairy farms in Michigan (n = 3) and Minnesota (n = 1). Cows were housed in freestalls and milked in parlors.

Cases were enrolled from June 2019 to March 2020 when it was prematurely ended due to the COVID-19 pandemic. Nonsevere cases of CM that occurred in a single quarter of cows not expected to dry off within 60 d and had not received any AM or had previous CM within 30 d were eligible. Trained farm workers aseptically collected milk samples (NMC, 2017) and recorded severity scores (Pinzón-Sánchez and Ruegg, 2011).

Duplicate milk samples were collected from eligible quarters, and one sample used to perform OFC while the second milk sample was frozen until picked up for testing at university laboratories. Trained employees used a sterile swab to inoculate approximately 0.1 mL of milk on a tri-plate (MN Easy Culture System; Laboratory for Udder Health, St. Paul, MN). Tri-plates included MacConkey agar (selective for GN growth), an agar selective for all GP bacteria and an agar selective for *Streptococcus*. After 24 h of incubation at 37°C, cases with ≥3 colonies of growth on GP agars were enrolled and randomly assigned to an experimental group. Farm workers were not aware which treatment would be assigned but were not blinded to treatments.

Quarters assigned to receive IMM AM were treated once daily using 1 of 3 protocols: 3 d of hetacillin potassium (**HP**, PolyMast, Boehringer Ingelheim, Duluth, GA), 3 d of ceftiofur hydrochloride (**CF**, Spectramast LC, Zoetis, Kalamazoo, MI), or 8 d of CF. Quarters assigned to the negative control group did not receive any treatments. Milk was discarded until it returned to normal (nontreated group) or until the end of AM withholding period after treatment. Researchers visited farms weekly to collect milk samples at 14, 21, and 28 ± 3 d after enrollment. Microbiological analysis in university laboratories was performed by inoculation of 10 µL of milk on 5% sheep blood agar and MacConkey agar (NMC, 2017). Isolates from plates with ≥3 cfu of GP growth were identified using MALDI-TOF at the Laboratory for Udder Health (University of Minnesota, Minneapolis, MN) and scores >2.0 were considered reliable.

Statistical analyses were conducted using SAS version 9.4 (SAS Institute Inc.). The experimental unit was quarter. Similar to Tomazi et al. (2021), we used unequal randomization (Dumville et al., 2006) because of previous research documenting the benefits of antimicrobial therapy (Oliver et al., 2004). Based on previously published distributions of pathogens and BC (Pinzón-Sánchez and Ruegg, 2011; Truchetti et al., 2014), enrollment of 190 quarters per group (α = 0.05; β = 0.80; difference of 10%) was originally planned. Enrollment ended prematurely due to prolonged research restrictions imposed by the COVID-19 pandemic. During this period, a revised estimate determined that enrollment of 20 cases in the nontreated group (**NT**) and at least 120 cases in a combined AM-treated group could detect a difference if BC was 50% in the NT group versus 80% in the combined AM treatment group (α = 0.05; β = 0.80).

Descriptive statistics were compared among groups using Fisher's exact test for categorical variables. Unadjusted crude proportions of BC were estimated for each sampling period using a subset of cases caused by target pathogens (*Staph.*, *Strep.*, and SLO). At each follow-up period, BC was defined as failure to isolate the same bacteria isolated at enrollment. Univariable associations were tested between risk factors and quarter-level BC at 21 d and covariates were eligible for entry to the multivariable model when *P* was <0.25.

Two multivariable logistic regression models were created using PROC GLIMMIX. One model evaluated the effect of experimental group (3-d IMM HP, 3-d IMM CF, 8-d IMM CF, no treatment) on BC at 21 d. The other model evaluated the combined effect of AM treatment as compared with the negative control at 21 d. In both final models, treatment was forced while selected variables were offered and evaluated using goodness-of-fit. Farm was included as a random effect in all models. Adjusted probabilities of outcomes were generated using LSMEANS and ODDS RATIO. Cases were omitted when any variables were missing. Risk factors considered for inclusion in both models included parity group (1 or 2+), severity at detection (mild or moderate), lactation stage (0–100, 101–200, >201 DIM), and season of enrollment (Jun–Aug or Sep–Mar). Risk factors were screened for 2- and 3-way interactions with treatment, but none were significant. In the combined model, subclinical mastitis history (**SCH**; log_10_ composite SCC 21–55 d before enrollment) was included as a continuous variable.

The 4 farms each contained about 900 to 10,000 lactating cows housed in freestalls and milked 3 times per day. Two farms milked Holsteins, 1 milked Holstein Jersey crossbreds, and 1 milked Jerseys. All farms used typical management practices such as pre- and postmilking teat disinfection, segregation of clinical cases, and standardized milking routines that included forestripping. Results of this study should be applicable to similar herds in the midwestern United States.

Among all farms, 256 cases of CM were enrolled but 16 cases from 1 farm were removed due to noncompliance, leaving 240 cases. The primary objective was to determine confirmed etiologies of CM cases treated based on growth of ≥3 cfu on GP segments of media used for OFC. Confirmed etiologies were verified using MALDI-TOF performed on bacteria isolated from duplicate milk samples collected at the same time. Of duplicate milk samples tested in university laboratories, no bacteria were grown from 15% of samples, and 7% were either contaminated or missing (Table 1). Based on MALDI-TOF used to identify bacteria grown from duplicate milk samples (Table 1), confirmed etiologies included *Strep.* spp. (n = 52; 22%), *Lacto.* spp. (n = 46; 19%), NAS (n = 39; 16%), other organisms (n = 24; 10%), *Staph. aureus* (n = 16; 7%), and *Entero.* spp. (n = 12; 5%). The distribution of pathogens did not vary among groups (*P* = 0.24) but did vary among farms (*P* = 0.001).

**Table 1:** Results of MALDI-TOF analysis to confirm identify isolates from duplicate milk samples collected from 240 cases of clinical mastitis enrolled in a trial based on identification as gram-positive organisms using selective agar from June 2019 to March 2020^1^

Organism	3-d HP	3-d CF	8-d CF	No treatment	Total	%^2^
*Streptococcus*-like organisms						
*Enterococcus*						5.0
*Enterococcus aquimarinus*	1	0	0	0	1	
*Enterococcus faecalis*	0	1	0	0	1	
*Enterococcus saccarolyticus*	3	2	3	2	10	
*Lactococcus*						19.2
*Lactococcus garvieae*	4	4	7	3	18	
*Lactococcus lactis*	14	6	7	1	28	
*Streptococcus*						21.7
*Streptococcus dysgalactiae*	7	13	7	5	32	
*Streptococcus uberis*	4	4	1	0	9	
*Streptococcus*spp.	3	2	4	2	11	
NAS						16.3
*Staphylococcus chromogenes*	2	8	8	2	20	
*Staphylococcus epidermidis*	0	0	1	0	1	
*Staphylococcus haemolyticus*	1	1	1	0	3	
*Staphylococcus simulans*	1	1	3	1	6	
*Staphylococcus*spp.	1	2	2	3	8	
*Staphylococcus xylosus*or *saprophyticus*	0	0	1	0	1	
*Staphylococcus aureus*						6.7
*Staph. aureus*	3	7	3	3	16	
Other	5	5	5	1	16	10.0
Mixed culture^3^	3	1	1	0	5	
Yeast	1	1	1	0	3	
No significant growth in duplicate sample^1^	12	6	11	6	35	14.6
Contaminated and missing	4	5	4	3	16	6.7
Total	69	69	70	32	240	

1 At enrollment duplicate quarter milk samples were collected. One sample was used for on-farm culture and the sample was enrolled based on those results. The second sample was frozen and used for MALDI-TOF.

2 Percentage of total enrolled cases.

3 Two causative pathogens were isolated from a mixed culture.

In other studies, NAS and *Strep.* are usually responsible for approximately one-third of all cases of CM, but these results have usually been determined based on classical microbiological testing in university laboratories (Oliveira and Ruegg, 2014). As researchers have increased use of MALDI-TOF, a broader range of GP organisms have been identified and efficacy data for IMM AM used to treat these organisms are lacking. Consistent with Scillieri-Smith et al. (2020), over 20% of our cases were caused by SLO. The tri-plates we used have been shown to have >80% accuracy for broad identification of *Staphylococcus* and environmental *Streptococcus* (Royster et al., 2014). However, no *Lacto.* or *Entero.* were included in that study and these organisms would appear phenotypically identical to *Strep.* using OFC. In a typical selective treatment protocol, IMM AM would be prescribed even though approved IMM AM have unknown efficacy against these organisms.

Farmers recorded the number of cfu on OFC agars and cases were enrolled only when ≥3 cfu were observed on GP agar. Of duplicate milk samples cultured in our laboratories, 15% had no significant bacterial growth, but discordant samples were randomly distributed among treatments (*P* = 0.54). Milk samples were typically frozen for <10 d before processing in university laboratories, but the freeze-thaw cycle may have reduced bacterial numbers to less than the detection limit and this may have occurred more commonly in milk samples that had sparse bacteria. Differences in inoculum volume between OFC and university laboratories may also have contributed to the discrepancy. Tri-plates used for OFC were inoculated using swabs that contain about 0.1 mL of milk, whereas agar plates in university laboratories were inoculated using loops containing 0.01 mL of milk. The number of cfu has been shown to be associated with the probability of BC (Deluyker et al., 2005); thus, fewer colonies detected in the original sample may have indicated these cases were more likely to achieve spontaneous BC without AM therapy.

Among enrolled cases (Table 1), 165 (70%) were caused by our target pathogens (*Strep.*, SLO, or *Staph.*). Of those cases, 78% of cows remained in the herd for all 3 follow-up samplings, although a few samples were lost, contaminated, or not collected at each sampling period (Table 2). Among the 3 follow-up dates, the overall (including treated and NT cases) crude BC for all cases varied slightly from 68% to 71% and were like values previously reported for positively controlled trials (McDougall et al., 2007). Crude BC ranged between 81% and 91% for cases confirmed as *Strep.*, 58% to 67% for cases confirmed as SLO, 79% to 80% for cases confirmed as NAS, and 33% to 43% for cases confirmed as *Staph. aureus* (Table 2). Within this subset of target pathogens, among sampling dates, the overall crude BC proportion of cases that received IMM AMs ranged from 69% to 71% (Table 2). The crude BC proportion of the small number of NT cases was 58% at 14 d and ranged from 67% to 73% by 21 to 28 d (Table 2). These values should be viewed cautiously due to the small number of cases and wide confidence intervals but indicate that NT animals can be safely included in future studies. Crude BC ranged from 56% to 63% for cases that received IMM HP and from 68% to 79% and 71% to 84% for cases that received 3 or 8 d of IMM CF, respectively (Table 2), which is consistent with previously reported values for cases caused by GP pathogens (Ruegg, 2021). While our study was not sufficiently powered to determine pathogen-specific effects, crude BC was numerically less for cases caused by SLO (Table 2). In vitro, susceptibility testing of our isolates indicated only 64% of SLO were susceptible to ampicillin (the active metabolite for HP), whereas 100% were susceptible to CF (Kolar et al., 2024). Thus, resistance to ampicillin may have influenced our results. However, susceptibility results should be viewed cautiously as there are no validated breakpoints for SLO and clinical outcomes of mastitis therapy are not strongly associated with results of in vitro susceptibly testing (Hoe and Ruegg, 2005).

**Table 2:** Descriptive results of bacteriological culture of milk samples collected at 14, 21, and 28 d for cases confirmed as *Streptococcus*, SLO, NAS, and *Staphylococcus aureus* that remained in the study for at least 28 d

Item	Days after enrollment in study			
		14 (95% CI)	21 (95% CI)	28 (95% CI)
Etiology at enrollment				
All cases	Total samples (n)	108	105	113
	Culture negative (n)	73	75	80
	Apparent BC^1^ (%)	67.6 (58–76)	71.4 (62–80)	70.8 (62–79)
Streptococci	Total samples (n)	25	21	23
	Culture negative (n)	21	17	21
	Apparent BC (%)	84.0 (64–95)	81.0 (58–95)	91.3 (72–99)
SLO^2^	Total samples (n)	47	48	54
	Culture negative (n)	27	32	33
	Apparent BC (%)	57.5 (42–71)	66.7 (52–80)	61.1 (47–74)
NAS	Total samples (n)	28	30	29
	Culture negative (n)	22	24	23
	Apparent BC (%)	78.6 (59–91)	80.0 (61–92)	79.3 (60–92)
*Staph. aureus*	Total samples (n)	8	6	7
	Culture negative (n)	3	2	3
	Apparent BC (%)	37.5 (9–76)	33.3 (4–78)	42.9 (9–82)
Experimental group				
All IMM treatments	Total samples (n)	96	94	101
	Culture negative (n)	66	67	72
	Apparent BC (%)	68.8 (58–78)	71.2 (61–80)	71.3 (61–80)
3 d IMM HP	Total samples (n)	34	30	35
	Culture negative (n)	19	19	19
	Apparent BC (%)	55.9 (38–73)	63.3 (44–80)	54.3 (37–71)
3 d IMM CF	Total samples (n)	31	29	33
	Culture negative (n)	21	23	26
	Apparent BC (%)	67.7 (49–83)	79.3 (60–92)	78.7 (61–91)
8 d IMM CF	Total samples (n)	31	35	33
	Culture negative (n)	26	25	27
	Apparent BC (%)	83.9 (66–94)	71.4 (54–85)	81.8 (61–93)
No treatment	Total samples (n)	12	11	12
	Culture negative (n)	7	8	8
	Apparent BC (%)	58.3 (28–85)	72.7 (39–94)	66.7 (35–90)

1 Bacteriological cure.

2 SLO include cases confirmed as *Lactococcus* spp. and *Enterococcus* spp.

Univariate associations between experimental group and risk factors for BC were assessed for 122 cases using culture results at enrollment and d 21. Estimates for BC varied less than we hypothesized and did not differ based on experimental group, season of enrollment, parity, or lactation stage (*P* > 0.40, Table 3). The final logistic regression model for the effect of experimental group on BC included only experimental group (*P* = 0.46) and severity (*P* = 0.04). As LSM for BC were similar, BC was compared between all treated cases and cases in the smaller NT group using a multivariable model with backward stepwise regression, with an additional risk factor of SCH 21 to 55 d before case onset. In that model, the proportion of BC at d 21 (LSM ± SE) did not vary between the combined cases that received AM (0.77 ± 0.06) and cases in the NT group (0.73 ± 0.16; *P* = 0.80), but these estimates lack precision and should be confirmed with larger studies. While there was a univariate association between mastitis severity and BC (Table 3), this risk factor did not remain in the final multivariable model (*P* = 0.28). However, the odds of primiparous cows experiencing BC by d 21 were 4.9× greater than for multiparous cows (*P* = 0.06; 95% CI: 0.93, 26.5) and each 1 log unit increase in log_10_SCC at the last test before the case decreased odds of BC by 1.3-fold (*P* = 0.005, 95% CI: −2.22, −0.42).

**Table 3:** Univariate associations between selected categorical risk factors and quarter-level bacteriological cure (BC) at 21 d after enrollment in a randomized clinical trial from June 2019 to March 2020

Predictor	BC (n = 122)			
	No.	%	95% CI	*P*-value
Experimental group				0.54
Negative control	08/12	66.7	38–88	
3 d IMM hetacillin	23/36	63.9	47–78	
3 d IMM ceftiofur	27/34	79.4	64–91	
8 d IMM ceftiofur	28/40	70.0	55–82	
Severity of CM				0.25
Mild	70/105	66.7	58–75	
Moderate	16/17	94.1	74–99	
Season of enrollment				0.93
Warm (Jun–Aug)	30/42	71.4	57–84	
Cool (Sep–Mar)	56/80	70.0	59–79	
Parity				0.17
1	19/24	79.2	60–91	
2+	67/98	45.4	59–77	
Lactation stage				0.48
0–100 (early)	44/63	69.8	58–80	
101–200 (mid)	30/39	76.9	61–88	
300+ (late)	12/20	60.0	38–79	

In the United States, most cases of mastitis are treated using IMM AM that are given for longer durations than listed on the approved product labels (Oliveira and Ruegg, 2014; de Campos et al., 2023). Limited research has evaluated the effect of treatment duration on BC using naturally occurring cases of CM. Longer duration treatments result in additional AM usage and greater costs (de Campos et al., 2023). Very few mastitis treatment trials have included NT cases caused by GP pathogens (Ruegg, 2021; Tomazi et al., 2021), and our results suggest that additional, negatively controlled, larger trials to evaluate the impact of treatment duration are warranted. Based on milk samples collected after 21 d, BC of cases that received longer duration IMM treatment (using CF) did not appear to be meaningfully different from cases that received shorter duration therapy. Previous researchers have examined benefits of longer duration treatment using experimentally induced *Strep. uberis* cases (Oliver et al., 2003) but have not included cases caused by SLO or NAS. Tomazi et al. (2021) included a NT group, but AM were given after 5 d, thus limiting inferences for the NT group. Although relatively few cases were included in our negative control group, our results provide preliminary evidence that can be used to justify future studies that include larger numbers of NT animals.

Although the primary emphasis of our study was to confirm the etiology of nonsevere cases of mastitis that were treated based on results of OFC, our results for BC are similar to results of previous positively controlled clinical trials (Truchetti et al., 2014). Although previous studies included relatively few cases caused by staphylococci, streptococci, or SLO, BC for those pathogen groups were similar to our values. Inclusion of negative controls allows determination of the additive effect of therapy relative to spontaneous BC. Additional negatively controlled studies that include larger number of cases caused by GP pathogens are needed to ensure that AM are used appropriately. The results we observed provide additional justification for these trials.

Our results confirmed the importance of several cow-level risk factors including parity and SCH on the likelihood of BC (Pinzón-Sánchez and Ruegg, 2011). As reported by others (Fuenzalida and Ruegg, 2019), primiparous cows had greater odds of BC. Similarly, cows with a history of greater SCC before CM were less likely to achieve BC, which is possibly a reflection of the duration of infection. One limitation was that we only sampled the clinically affected quarter, and did not evaluate the adjacent quarters, thus leaving a potential source of reinfection. We encourage producers to review SCC history and parity to help make informed decisions about treatment of individual animals.

Nonsevere CM identified based on growth on GP agars used for OFC was caused by a diverse group of pathogens, many of which lack data about treatment efficacy. Among these pathogens, proportions of cases that achieved BC were similar to previous studies and did not vary among experimental groups. These results support reviewing parity and SCC before making treatment decisions, as mild cases of CM and CM in primiparous animals without a history of subclinical mastitis were more likely to achieve BC. Additional negatively controlled clinical trials evaluating use of IMM AM for GP cases of nonsevere CM can be safely conducted and are needed to determine appropriate duration and usage of IMM AM therapy.
